# Pharmaceutical Co-Crystal Formulation of Rivaroxaban with Niacinamide: Preparation, Characterization, and In Vitro Release Evaluation

**DOI:** 10.3390/ma19071336

**Published:** 2026-03-27

**Authors:** Cristina Solomon, Iulian Sarbu, Valentina Anuța, Emma Adriana Ozon, Adina Magdalena Musuc, Adriana Rusu, Vasile-Adrian Surdu, Abhay Chandak, Roxana Mariuca Gavriloaia, Ancuța Cătălina Fița, Denisa Teodora Nită, Mirela Adriana Mitu

**Affiliations:** 1 Faculty of Pharmacy, “Carol Davila” University of Medicine and Pharmacy, 6 Traian Vuia St., 020956 Bucharest, Romania; cristina.solomon@drd.umfcd.ro (C.S.); emma.budura@umfcd.ro (E.A.O.); catalina.fita@umfcd.ro (A.C.F.); denisa-teodora.nita0720@stud.umfcd.ro (D.T.N.); mirela.mitu@umfcd.ro (M.A.M.); 2 Faculty of Pharmacy, “Titu Maiorescu” University, 004051 Bucharest, Romania; iulian.sarbu@prof.utm.ro (I.S.); roxana.gavriloaia@prof.utm.ro (R.M.G.); 3 Innovative Therapeutic Structures Research and Development Centre (InnoTher), “Carol Davila” University of Medicine and Pharmacy, 6 Traian Vuia Street, 020956 Bucharest, Romania; valentina.anuta@umfcd.ro; 4 Institute of Physical Chemistry—Ilie Murgulescu, Romanian Academy, 060021 Bucharest, Romania; arusu@icf.ro; 5 Department of Materials Science, Faculty of Materials Science and Engineering, Transilvania University of Brasov, 29 Eroilor Blvd., 500036 Brasov, Romania; vasile.surdu@unitbv.ro; 6 Zentiva Group, U Kabelovny 529/16, 102 00 Prague-Dolní Měcholupy, Czech Republic; abhaykumar.chandak@zentiva.com

**Keywords:** rivaroxaban, niacinamide, pharmaceutical co-crystals, dissolution enhancement, co-crystallization

## Abstract

The present study investigates the co-crystallization process of rivaroxaban (RIV), a poorly water-soluble potent oral anticoagulant, with niacinamide (NIA), a highly soluble and pharmaceutically acceptable co-crystal former, in two different molar ratios (1:1 and 1:2). The aim was to enhance the physicochemical and biopharmaceutical properties of rivaroxaban such as dissolution rate and aqueous solubility, by forming stable co-crystals through a solvent evaporation technique. The resulting co-crystals (RIV-NIA, 1:1 co-crystallization compound, F1 and RIV-NIA, 1:2 co-crystallization compound, F3) were characterized using scanning electron microscopy (SEM), Fourier-transform infrared spectroscopy (FTIR), powder X-ray diffraction (XRD) and thermal analysis, which confirmed the formation of a new rivaroxaban–niacinamide co-crystalline phase. In vitro dissolution studies confirmed a significant enhancement in the dissolution rate of the two obtained co-crystals. These findings suggest that stoichiometric variation plays an important role in co-crystal performance and in improving solubility compared with the pure drug. Also, the obtained results suggest that niacinamide is an effective coformer for improving the dissolution and physicochemical properties of rivaroxaban.

## 1. Introduction

Enhancing the solubility, dissolution profile, stability, pharmacokinetics, and bioavailability of soluble drugs with lower solubility in water is a permanent challenge in pharmaceutical science. Among the various strategies employed to address these issues, the co-crystallization technique has gained significant consideration as a solid-state approach able to modify the physical, chemical, and pharmacotechnical properties of active pharmaceutical ingredients (APIs) without compromising their efficiency. Pharmaceutical co-crystals are supramolecular crystalline solids composed of API and a neutral substance acceptable for pharmaceutical use, such as former [1] or coformer [2], both existing in the solid form at room temperature [3]. These co-crystals are defined as crystals formed by two or more distinct molecules (from which one is the active pharmaceutical ingredient) at a stoichiometric ratio that are formed through various noncovalent intermolecular interactions, including van der Waals forces, halogen bonding, π–π stacking interactions, and most commonly, hydrogen bonding [4]. The co-crystal not only exhibits different physicochemical properties [5], but also differs in its crystalline structure from its individual components [6]. The supramolecular structures created by non-covalent bonding of identical functional groups (homosynthons) and different functional groups (heterosynthons) can be used to explain the molecular arrangement of co-crystals [7].

Recently, a growing interest in the design and development of pharmaceutical co-crystals due to their potential to enhance the solubility, thermal stability, and dissolution rate of poorly soluble drugs without altering their molecular structure has increased [8]. The challenges associated with the low solubility of active substances belonging to class II of the Biopharmaceutical Classification System (BCS) are a major concern in pharmaceutical research, and rivaroxaban (RIV) is a relevant example in this regard. The specialized literature describes numerous strategies to improve the solubility and dissolution rate of RIV, including solid dispersions, nanometric systems, lipid formulations, or amorphous forms. Although these approaches can lead to significant increases in dissolution, they are often accompanied by limitations such as physical instability, tendency to recrystallize, use of polymeric excipients in large quantities or complex technological processes, and difficulty in scaling up industrially.

**Figure 1:**
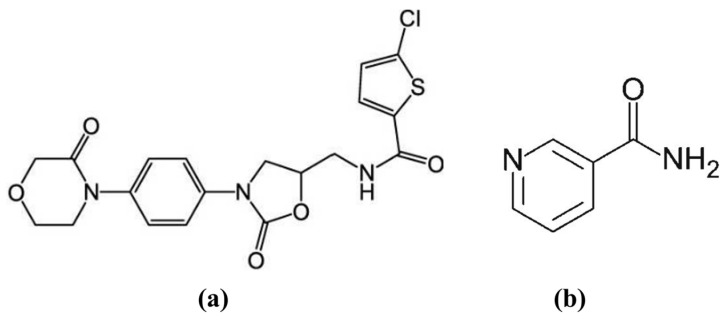
Chemical structure of (**a**) rivaroxaban (RIV) and (**b**) niacinamide (NIA).

Rivaroxaban (RIV) (Figure 1a), an oral anticoagulant and a selective direct Factor Xa inhibitor, has low aqueous solubility, which limits its absorption and therapeutic performance, and is therefore classified as a Biopharmaceutics Classification System (BCS) Class II drug. RIV is frequently used in the treatment and prevention of venous thromboembolism, stroke in atrial fibrillation, and other thrombotic conditions [9]. By modifying its solid-state form through co-crystallization, a promising approach to improve its dissolution characteristics can be obtained, which is essential for achieving consistent and optimal therapeutic efficiency. Literature studies have demonstrated the feasibility of forming co-crystals with rivaroxaban; however, the influence of stoichiometric variation in drug–co-former molar ratios has not been sufficiently explored [10].

Among various co-formers, niacinamide or nicotinamide (NIA), also known as pyridine-3-carboxamide—a water-soluble form of vitamin B3 (Figure 1b), is frequently used as a pharmaceutical co-former due to its excellent safety profile, strong hydrogen bonding donor/acceptor capability, biocompatibility, low molecular weight, and its ability to enhance the pharmacokinetic properties and water solubility of co-formulated drugs compared to the API alone [11,12]. Compared with previously reported formulations, the novelty of the research is distinguished by the introduction of a novel pharmaceutical co-crystal of rivaroxaban with niacinamide, a co-former with a well-known and regulatory-acceptable safety profile. Unlike polymeric excipients or surfactants commonly used in other formulation strategies, niacinamide is a low molecular weight molecule capable of forming stable hydrogen bond networks, favoring the achievement of a well-defined and reproducible crystal structure.

The present study aims to synthesize and characterize two rivaroxaban–niacinamide co-crystals prepared at different molar ratios (1:1, named as F1 and 1:2, named as F3) using the “solvent evaporation” method to evaluate their solid-state properties. The novelty lies in the complementary investigation of the structural, thermal, and morphological properties of the resulting co-crystals. This research evaluates how the molar ratio influences the nature of the solid forms, the assessment of the potential of niacinamide to enhance the dissolution behavior of rivaroxaban due to limited studies, and the possible pharmaceutical advantages. Advanced analytical techniques, including Fourier-transformed FT-IR spectroscopy, scanning electron microscopy (SEM), X-ray powder diffraction (XRD), and thermal analysis (TG–DTA), were employed to clarify the physicochemical profile of both co-crystal systems. Furthermore, measurements such as flowability, bulk and tapped densities, and compressibility index were carried out to evaluate their pharmacotechnical properties [13]. Finally, in vitro dissolution experiments were performed in two different simulated gastrointestinal media to evaluate the dissolution profiles of the co-crystals with those of pure rivaroxaban.

## 2. Materials and Methods

### 2.1. Materials

Niacinamide was obtained from Fagron (Trikala, Greece) and micronized RIV (Form I), manufactured by Neuland Laboratories Limited, was donated by Labormed-Pharma SA, Romania. Gradient-grade acetonitrile, which is appropriate for HPLC, was purchased from Merck KGaA (Darmstadt, Germany). Sodium hydroxide (extra pure, 50 wt% solution in water) and formic acid (99.0+%, Optima™ LC/MS Grade) were acquired from Fisher Chemical (Thermo Fisher Scientific, Waltham, MA, USA). Using a Milli-Q EQ 7008 purification system (Merck Millipore, Burlington, MA, USA), ultrapure water (18.2 MΩ·cm at 25 °C) was produced. Merck KGaA (Darmstadt, Germany) provided other reagents, such as potassium phosphate monobasic, sodium acetate, acetic acid, and sodium dodecyl sulphate.

All chemicals and solvents used were of analytical reagent grade.

### 2.2. Methods

#### 2.2.1. Preparation of the RIV-NIA Samples

In the study, co-crystals formed by RIV with NIA in two molar ratios, 1:1 for F1 and 1:2 for F2 (RIV:NIA) were prepared, and simple physical mixtures (1:1 molar ratio for F3 and 1:2 molar ratio for F4) were used as reference samples for the characterization studies of the co-crystals.

##### Preparation of the RIV-NIA Co-Crystal

The “solvent evaporation” method was selected as the co-crystal technology for RIV-NIA systems. 0.5 g RIV and 0.14 g NIA for 1:1 molar ratio and 0.5 g RIV and 0.28 g NIA for 1:2 molar ratio were ground and mixed in a mortar with a pestle, then acetone was slowly added until the mixture was completely dissolved. The solvent was evaporated in a Stuart RE300 rotary evaporator from Bibby Scientific, France. The mixture was then allowed to dry at room temperature for 20 h.

White, homogeneous, and fine powders were obtained.

##### Preparation of the RIV-NIA Physical Mixtures

The physical mixtures were prepared by simply mixing the RIV with NIA for 2 min at room temperature.

#### 2.2.2. Physicochemical Characterization of the RIV-NIA Systems

*The FTIR measurements* were conducted in transmission mode using a NICOLET 6700 FT-IR spectrophotometer (Thermo Electron Corporation, Waltham, MA, USA). The FTIR spectra were collected in the range of 4000–400 cm^−1^, with a resolution of 4 cm^−1^. The samples were prepared as thin, transparent KBr pellets (20 mg/cm^2^) with approximately 0.5% of the sample. Each pellet was formed by thoroughly grinding 1 mg of the sample with 200 mg of KBr, followed by compression under vacuum to ensure homogeneity.

*Thermal analysis* was made with a NETZSCH STA 449 F3 Jupiter instrument (Selb, Germany), in the temperature range of 25–600 °C in alumina crucibles. Thermogravimetric (TG) and Differential Thermal Analysis (DTA) curves were acquired in nitrogen, atmosphere at a flow rate of 20 mL/min and a 10 °C/min heating rate. The experimental data were processed using NETZSCH Proteus—Thermal Analysis software, version 5.2.1.

*X-ray diffraction (XRD)* measurements were made using a Bruker D8 Advance diffractometer, utilizing Ni-filtered Cu-Kα radiation (λ = 1.5418 Å) equipped with an X-ray tube that operated at 40 kV and 40 mA. On the incident beam side, motorized slits with a 0.25 mm aperture and a 2.5° Soller slit were used. On the diffracted beam side, 5 mm motorized slits were attached to a LYNXEYE XE-T detector, functioning in 1D high-resolution mode. XRD patterns were collected over a 2θ range of 5–60°, with a step size of 0.02° and a counting time of 0.2 s per step. Phase composition was analyzed in HighScorePlus 3.0.e software coupled with COD database. The refinement of the patterns was carried out by Rietveld formalism [14] using a polynomial function for background, a pseudo-Voigt function for peak profile, and a Caglioti function for peak width approximation.

*Morphological analysis* (SEM) of the raw materials and resulting co-crystal compounds was performed using a Tescan Vega LMU Scanning Electron Microscope (from Brno, Czech Republic). The equipment operated in low vacuum mode (20 Pa) at an accelerating voltage of 10 kV.

#### 2.2.3. Development and Manufacturing of the Oral Tablets

##### Formulation of the Tablets

Based on the results of the preformulation studies, four series of tablets containing the RIV-NIA co-crystallization compounds were formulated [13].

The oral tablet formulations are shown in Table 1.

##### Manufacturing Process

The materials were compressed with varying compression forces as needed (10 kN for F1 and F3, 6 kN for F2, and 8.5 kN for F4) in a single-post eccentric tablet press (Erweka EP-1 from Erweka, Langen, Germany). The machine was equipped with 10 mm flat punches and set up for the production of 200 mg tablets.

#### 2.2.4. Quality Attributes of the Tablets

##### Organoleptic Properties

The tablets’ appearance was evaluated in accordance with the European Pharmacopoeia specifications [15].

##### Dimensions (Diameter and Thickness)

Ten tablets of each batch were tested for thickness and diameter using a VK 200 tablet hardness tester from Vanderkamp, New York, NY, USA.

##### Mass Uniformity

Each formulation’s twenty tablets were weighed separately, and the average weight was determined [15].

##### Hardness

The hardness was measured using a VK 200 tablet hardness tester. It is stated as the amount of force needed to crush the tablets that are placed between the device’s two anvils. Each batch’s ten tablets were examined.

##### Friability

Ten tablets from each series were analyzed with the Vankel friabilator. The tablets were weighed, then put into the device’s drums and spun at 30 rpm for five minutes. The tablets were dedusted and weighed once more to calculate the mass loss during rotation. The upper limit, as per compendial norms, is 1.0% [15].

##### In Vitro Disintegration Time

Six tablets of each formulation were tested for disintegration behavior according to European Pharmacopoeia standards in distilled water at 37 ± 0.5 °C [15]. The time in seconds needed for complete disintegration was measured using an Erweka DT 3 equipment, which is produced by Erweka^®^ GmbH in Langen, Germany.

##### In Vitro Release Study

A Vision G2 Classic 6 Dissolution Tester (Teledyne Hanson, Chatsworth, CA, USA) equipped with a USP Apparatus II (paddles) was used to evaluate the drug release profiles of the 10 mg rivaroxaban tablets. Dissolution was performed at 37.0 ± 0.5 °C and 75 rpm according to USP recommendations for rivaroxaban tablets [16]. Two different media were tested in each vial containing 900 mL of dissolution material: a 0.05 M phosphate buffer with a pH of 6.8 without surfactants and a 0.022 M sodium acetate buffer with a pH of 4.5 and 0.2% sodium dodecyl sulphate (the medium recommended for 10 mg rivaroxaban tablets).

At 5, 10, 15, 20, 30, 45, 60, 90, 120, and 180 min, aliquots of 1.5 ± 0.1 mL were taken out. To maintain the sink conditions, an equivalent volume of newly heated medium was injected after each withdrawal. Before analysis, a 0.45 μm polyethersulfone membrane was used to filter the obtained samples. Each dissolution run was carried out three times. The same dissolution experiments were also conducted on rivaroxaban alone, used as a reference product, to provide a more transparent evaluation of the drug release performance.

##### HPLC Analysis

Rivaroxaban quantification was carried out using a validated reversed-phase HPLC method that was adapted from a previously reported technique [17]. Chromatographic separations were performed using a 100 × 3 mm Kinetex^®^ C18 column (2.6 µm particle size, Phenomenex, Torrance, CA, USA) kept at 45 °C in a Jasco 4000 Series HPLC system (JASCO Corporation, Tokyo, Japan). 0.1% formic acid (A) and acetonitrile (B), combined in a 62:38 (*v*/*v*) ratio, made up the mobile phase. 250 nm was the UV detection setting. Following the current ICH criteria [18], the HPLC test was validated to guarantee linearity, accuracy, precision, specificity, and sensitivity. To accurately determine the dissolved drug fraction in each sample, standard solutions of rivaroxaban were prepared throughout a concentration range of 0.156–20 µg/mL for calibration curves.

## 3. Results and Discussion

### 3.1. Physicochemical Characterization

#### 3.1.1. FTIR Analysis

**Figure 2:**
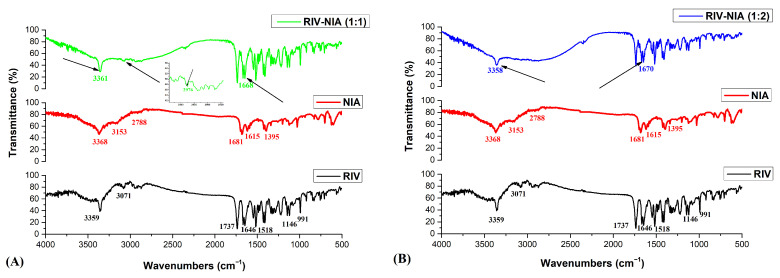
(**A**) FTIR diagrams of compounds: rivaroxaban (RIV) (black line), niacinamide (NIA) (red line), RIV-NIA (1:1) (green line), and (**B**) FTIR diagrams of compounds: rivaroxaban (RIV) (black line), niacinamide (NIA) (red line), RIV-NIA (1:2) (blue line).

FTIR analysis was used to identify the intermolecular interactions and changes in the bonding of functional groups. Figure 2A,B represents the FTIR analysis of raw materials (rivaroxaban and niacinamide) and the two obtained co-crystals (1:1 and 1:2 molar ratios). The FTIR spectrum of RIV (black line from Figure 2) showed characteristic absorption bands at 3359 cm^−1^ characteristic for secondary amide N-H group stretching, 1737 cm^−1^ due to carbonyl C=O stretching from the ester group, 1646 cm^−1^ due to amide C=O stretching, 1518 cm^−1^ due to aromatic C=C stretching, 1146 cm^−1^ due to the corresponding C-O-C movement presents in both esters and ethers, 991 cm^−1^ due to carbonyl C-H bending, and between 850 and 550 cm^−1^ due to C-Cl stretching [19]. The obtained FTIR spectrum is in agreement with literature-reported data of rivaroxaban form I spectrum [20].

The FTIR spectrum of NIA (red line from Figure 2) had peaks corresponding to N-H symmetric and asymmetric stretching vibrations at 3153 and 3368 cm^−1^, at 2788 cm^−1^ peak corresponding to C-H aromatic stretching vibration, a peak at 1681 cm^−1^ corresponding to C=O stretching vibration, a peak at 1615 cm^−1^ corresponding to C=C aromatic stretching vibration and at 1395 cm^−1^ which corresponds to C-N stretching vibration [21,22].

The FTIR spectra of RIV-NIA co-crystals shown in Figure 2A (green line for 1:1 molar ratio and blue line for 1:2 molar ratio from Figure 2B) show the following feature: the C=O stretching of RIV is shifted from 1646 cm^−1^ to 1668 cm^−1^ for RIV-NIA (1:1 co-crystallization compound), and to 1670 cm^−1^ for RIV-NIA (1:2 co-crystallization compound), respectively. The amino stretching of NIA is shifted from 3368 cm^−1^ to 3361 cm^−1^ for RIV-NIA (1:1 co-crystallization compound), and to 3358 cm^−1^ for RIV-NIA (1:2 co-crystallization compound), respectively. These shifts in positions of wavenumber to the higher values for RIV and the lower for NIA indicate that the new molecular interactions that take place in the RIV-NIA systems significantly influence the positions of the functional group. These modifications are due to the intermolecular interactions of each co-crystal [19,23].

In the FTIR spectra of the two RIV-NIA co-crystals, the presented shifts in the characteristic bands compared to the individual components indicate changes in the local hydrogen-bonding environment caused by the formation of new intermolecular interactions consistent with cocrystallization. In the RIV-NIA 1:1 cocrystal, the moderate shifts suggest the formation of a primary interaction between one API molecule and one coformer molecule. In contrast, the RIV-NIA 1:2 cocrystal exhibits more pronounced shifts, consistent with the involvement of a second coformer molecule participating in hydrogen-bonding interactions. The differences between the two stoichiometries support the formation of distinct supramolecular arrangements and highlight the influence of excess coformer on the interaction network within the solid state. Nevertheless, similar FTIR behavior has been reported in the literature for rivaroxaban-related multicomponent systems, where comparable IR absorption band shifts were attributed to hydrogen bonding interactions, due to intermolecular interactions of co-crystal [24].

#### 3.1.2. SEM Analysis

**Figure 3:**
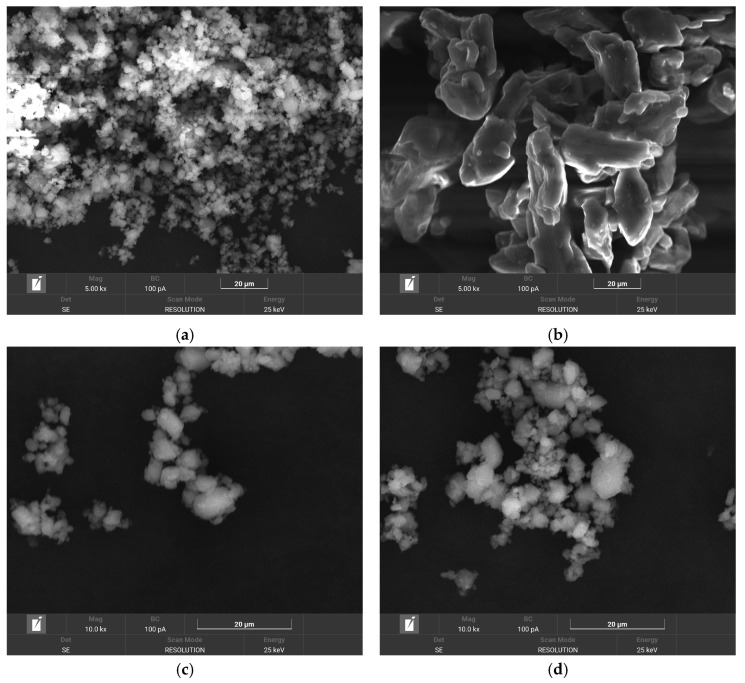
SEM images of (**a**) rivaroxaban (RIV), (**b**) niacinamide (NIA), (**c**) RIV-NIA (1:1 co-crystallization compound), (**d**) RIV-NIA (1:2 co-crystallization compound).

The morphologies of the RIV, NIA, and the prepared RIV-NIA (1:1 co-crystallization compound) and RIV-NIA (1:2 co-crystallization compound) are shown in Figure 3. The SEM image of RIV (Figure 3a) shows regular-shaped, nearly spherical crystals [25], and NIA (Figure 3b) shows pebble-shaped crystals [26]. In contrast, the two formulated co-crystals (Figure 3c,d) showed asymmetrical-to-spherical crystals with irregular surfaces. However, the morphologies of the two RIV-NIA co-crystals (1:1 and 1:2 molar ratios) were totally different from those of RIV and NIA raw materials. Further, this indicates that the obtained co-crystallization compounds had a new crystal state obtained under the effect of the solvent evaporation method.

#### 3.1.3. X-Ray Diffraction Analysis

**Figure 4:**
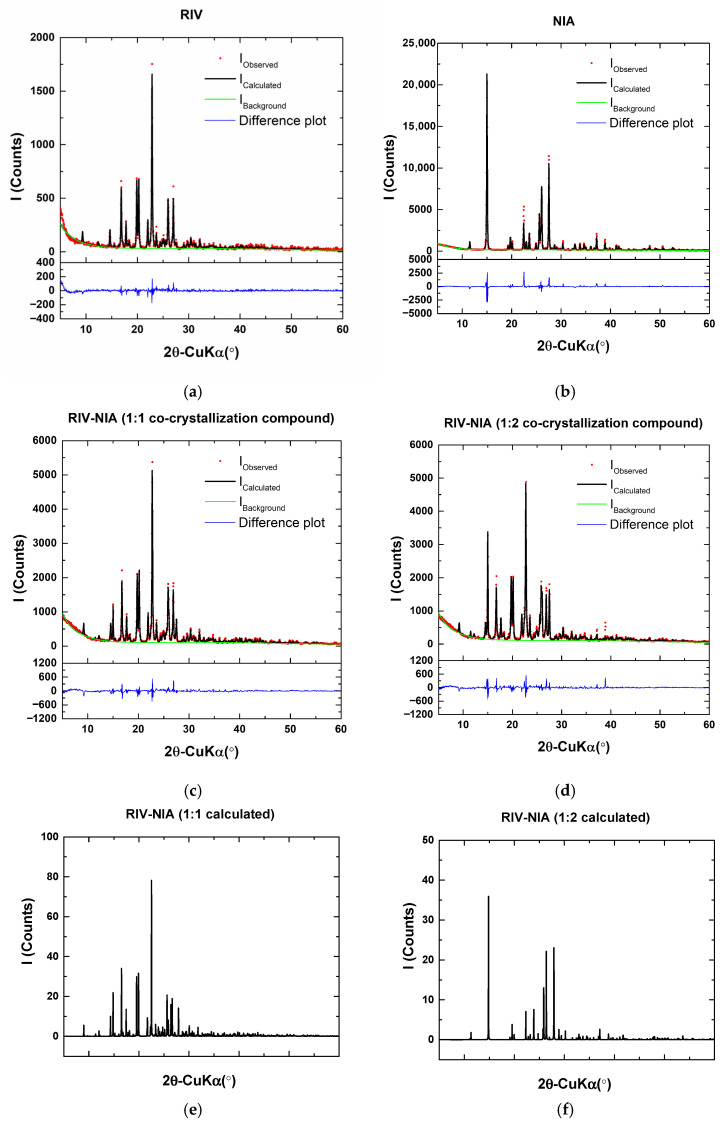
XRD diffractograms of compounds: rivaroxaban (RIV) (**a**), niacinamide (NIA) (**b**), RIV-NIA (1:1 co-crystallization compound) (**c**), RIV-NIA (1:2 co-crystallization compound) (**d**), RIV-NIA (1:1 calculated profile) (**e**), and RIV-NIA (1:2 calculated profile) (**f**).

XRD analysis was used to characterize the crystal structures of the powder compounds. Phase search and match revealed that the patterns can be identified with rivaroxaban (COD # 2,242,344 [27]) and niacinamide (COD # 2,003,051 [28]). The RIV and NIA XRD patterns show the most intense peaks located at 22.8568, 20.2734, 16.8682, 19.7252, and 27.0030, and respectively, 15.0058, 27.5267, 26.0620, 25.5993, and 23.5736° 2θ, which are shifted towards higher 2θ angles compared to the ones shown in the COD files (Figure 4).

The peaks observed for RIV-NIA 1:1 and 1:2 co-crystal compounds show decreases in the 2θ angle values compared to the pristine RIV and NIA. The comparison of the 2θ angles for the first five most intense peaks is summarized in Table 2 and Table 3. These features provide arguments for the successful co-crystallization process.

The difference and the peak intensities for the RIV-NIA co-crystal compounds as well as the peak shifts towards lower 2θ angles, indicate the formation of a new rivaroxaban–niacinamide co-crystalline phase. The weight fractions for rivaroxaban and niacinamide determined from Rietveld refinement of the patterns (Table 4) show 13.3% rivaroxaban and 86.7% niacinamide for RIV-NIA (1:1 cocrystallization compound) and, respectively, 30% rivaroxaban and 70% niacinamide for RIV-NIA (1:2 cocrystallization compound). In addition, the determined crystallinity (Table 4) of the samples is 59.29% and 61.66% for RIV-NIA (1:1 cocrystallization compound) and, respectively, RIV-NIA (1:2 cocrystallization compound). The differences between the nominal compositions and those determined by the refinement of the XRD patterns can be explained by the amorphous content.

The powder XRD patterns show the appearance of new diffraction peaks and the disappearance of characteristic reflections of the individual components, indicating the formation of a new solid phase consistent with a rivaroxaban–niacinamide co-crystal.

#### 3.1.4. Thermal Analysis

**Figure 5:**
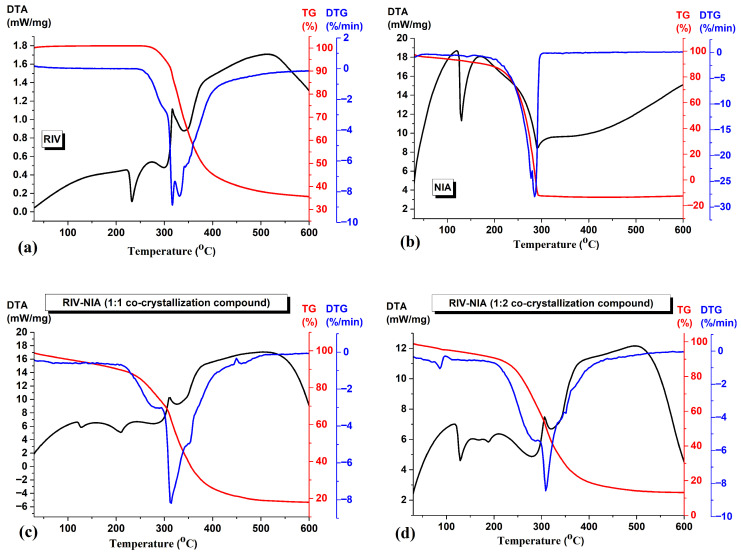
Thermal curves of (**a**) rivaroxaban (RIV), (**b**) niacinamide (NIA), (**c**) RIV-NIA (1:1 co-crystallization compound), (**d**) RIV-NIA (1:2 co-crystallization compound).

The thermogravimetric curves of the studied compounds are shown in Figure 5: (a) rivaroxaban (RIV), (b) niacinamide (NIA), (c) RIV-NIA (1:1 co-crystallization compound), (d) RIV-NIA (1:2 co-crystallization compound).

The TG-DTA curve of RIV (Figure 5a) shows the melting peak at 232.6 °C (on DTA curve), followed by the decomposition process with two maxima at 316.6 and 512.5 °C (from DTA curve) and 318.8 and 339.8 °C (from DTG curve) [29]. The final residue at 600 °C is about 35.6%. The TG-DTA curve of NIA (Figure 5b) shows the melting point at 129.3 °C, after which it starts to lose its weight between 200 and 300 °C, with peak temperature on DTA curve at 291.1 °C and on DTG curve at 282.6 °C, and a shoulder at 277.6 °C. The co-crystals prepared by the solvent evaporation method show several features:

(i) Both RIV-NIA (1:1 co-crystallization compound) (Figure 5c) and RIV-NIA (1:2 co-crystallization compound) (Figure 5d) show two endothermic events on DTA curve at 126.8 °C and 209.3 °C for co-crystal 1:1 molar ratio and 128.8 °C and 210.1 °C for co-crystal 1:2 molar ratio, respectively.
(ii) The first endothermal event, close to the melting peak of pure NIA, may be attributed to minor amorphous content within the multicomponent system, as XRD analysis confirms. There is more pronounced effect for co-crystal 1:2 molar ratio compared with co-crystal with 1:1 molar ratio; This is evidence for the higher content of niacinamide.
(iii) The second endothermic event, occurring at around 209 °C, corresponds to the melting of the newly formed multicomponent crystalline phase, indicating that a distinct solid phase has formed. These thermal observations are consistent with the XRD patterns, as well as with FTIR and SEM analyses, which further confirm the formation of a new co-crystalline system.
(iv) The shifting of the endothermal events in the co-crystallization compounds is a thermodynamic property of those, which indicates that the RIV-NIA should be in a new solid crystalline phase instead of a mixture.
(v) The co-crystals start to lose their weight after 250 °C, at a temperature higher than the individual components, in a complex decomposition step. The final residue at 600 °C is 17% for RIV-NIA (1:1 co-crystallization compound) and 13.2% for RIV-NIA (1:2 co-crystallization compound).

Melting point, as a fundamental physical characteristic, reflects the energy necessary to overcome the intermolecular forces maintaining the integrity of the crystal lattice. A diversity of factors, such as the chemical and crystal structure, molecular interactions, symmetry, and conformational flexibility, can influence this thermal property. Typically, enhanced molecular symmetry or the presence of strong hydrogen bonds correlates with increased intermolecular cohesion in the solid state, thus raising the melting point [21]. By analyzing the melting points of 50 documented pharmaceutical co-crystals, according to Schultheiss and Newman, it was observed that approximately half exhibited melting points within the range defined by their constituent API and conformer and around 40% of the co-crystals had melting points lower than those of either individual component [30].

Spectroscopic characteristics, morphological analysis, and thermal profiles of both RIV-NIA co-crystals described by FTIR, SEM, XRD, and TGA analyses demonstrate that new structures were obtained after the co-crystallization process.

### 3.2. Quality Attributes of the Tablets

**Figure 6:**
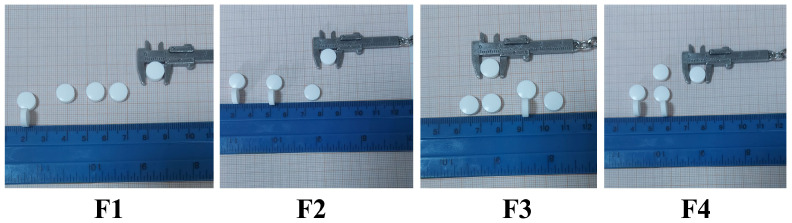
Tablets’ appearance.

The tablets are round, white, with a smooth surface and uniform in appearance (Figure 6).

The tablets’ properties of the four batches are shown in Table 5 [31].

The dimensions (diameter and thickness) and mass of the tablets varied within narrow ranges, indicating that the dies were filled evenly as the materials were sufficiently flowable. The good compactability of the materials and the correct setting of all parameters also contributed to the successful completion of the pressing process. Minor variations within and between the tablet series demonstrate that the size and mass of the tablets are not influenced by the type of active ingredient, but rather by the excipients. The weight of tablets in each of the four formulations is approximately 200 mg. The formulations have a diameter of 10 mm, and are 2.52–2.59 mm thick. These values correspond to the criteria of the European Pharmacopoeia [15] and show that the excipients and the compression conditions were chosen correctly. Achieving a homogeneous mass and size guarantees that the tablets have a uniform and adequate dosage.

The use of Avicel^®^ PH 102 (microcrystalline cellulose) as a filler leads to an increase in the range of particle binding due to plastic deformation during compression. It has also been suggested that elongated and irregularly shaped microcrystalline cellulose particles can be mechanically bonded together to improve compressibility [32,33]. The main reason for the remarkable binding properties of microcrystalline cellulose is its plasticity. The strain rate sensitivity (SRS) of microcrystalline cellulose, i.e., the greater elastic effects at higher compression speeds where there is insufficient time for plastic deformation, is also explained by its viscoelastic property. Due to its extremely low coefficient of friction and its extremely low residual pressure on the die wall [34,35], microcrystalline cellulose is a self-disintegrating binder [36] with low requirements on the lubricant in terms of its dry binding capacity. However, when using microcrystalline cellulose in a tablet formulation, these properties do not replace the addition of real disintegrants and lubricants. Microcrystalline cellulose reduces production loss and increases manufacturing efficiency by improving the compactability of the powder and facilitating dust-free handling during direct compression.

It can effectively bind other materials in low quantities, particularly active ingredients that are difficult to compress. While the wide variety of particle sizes offers the best packing density and coverage of other materials, microcrystalline cellulose has a high dilution potential [37]. Due to its low bulk density, it is the most popular filler. According to Ilić I. et al. [38], coverage of the active ingredient and other excipients, optimal packing density, and high dilution potential on a weight basis are all characteristics of an excipient with a large particle size distribution and low bulk density. In view of the aforementioned properties of microcrystalline cellulose, the uniformity of tablet dimensions and mass proves the correct choice of filler in all formulations.

The hardness, on the other hand, differs considerably between the individual batches, which indicates that the materials can be compressed to different degrees. The hardness ranges from 71 N to 90 N, and the batches with the same molar ratio of RIV and NIA differ significantly from each other. Since the same excipients are present in the same proportions in all formulations, the molar ratio and the method used to prepare the binary system have a significant influence on the elasticity and plasticity of the compounds. In all cases, the tablets with the simple physical mixture of ingredients have a higher strength (89 N for F2 and 90 for F4) than the formulations with the corresponding co-crystallization compounds (78 N for F1 and 71 N for F3). All tablets exhibited sufficient mechanical strength in terms of hardness and friability, although the formulations differed significantly.

The tablets’ friability does not vary essentially, and all values fall within the European Pharmacopoeia limit (<1.0%) [15].

The compression force used during tableting plastically deforms all of the particles, causing them to be extensively distributed throughout the tablet and enhancing its strength.

According to the findings of Cabiscol et al. [39], microcrystalline cellulose produces robust tablets with a slightly higher strength, whereas materials with a more brittle behavior (lactose) create structures that are about four times less strong at the same compression force. Although magnesium stearate greatly improves the flowability of the powders from the cone to the die, it weakens the hardness and tensile strength of the tablets. The strength of the tablet is determined by the region of close particle contact and the forces of attraction throughout the entire contacting area. The strength of the tablets can be decreased if the fine lubricant particles obstruct the interaction bonding forces between the particles to be compacted [40]. Since all formulations contain the same amount of magnesium stearate, it is clear that the differences in hardness are not caused by it, but by other ingredients contained.

Cabiscol R. et al. [39] also proved that microcrystalline cellulose shows a greater degree of axial elastic recovery when compared to lactose. This is ascribed to the elasto-plastic densification process of microcrystalline cellulose, in which the relaxation kinetics are determined by the combined competence of total elastic recovery and Poisson’s effect.

The time-dependent elastic expansion of the final tablet is integrated into the elastic recovery, which should occur in all directions. In the initial phase of compression, a normal force vector presses axially on the powder, which tends to grow perpendicularly when a uniaxial force is applied in the die. In the intermediate phases of compression, the free spaces shrink rapidly due to the entrapment in the die. Depending on the mechanical behavior of the raw material particles in a later phase of compression, this can lead to a variety of opposing effects, such as plastic deformation, which creates large interfaces between the particles in the case of ductile materials, or particle fragmentation in the case of brittle materials. Particles of microcrystalline cellulose tend to return to their original shape after compression, causing the tablet to expand axially and shrink radially [41,42].

On the other hand, the continuous fragmentation of lactose produces more microscopically small particles that fill the gaps left by larger particle packings and at the same time have a larger specific surface area. The more densely the particles are packed, the more particle–particle contact areas are created, but these do not contribute to the surface area [43].

It is known that the disintegration time varies indirectly proportional to the hardness and tablets with high mechanical strength probably also have a high disintegration time [44]. Unexpectedly, the disintegration time is highly dependent on the molar ratio between RIV and NIA and not dependent on the hardness. Nevertheless, there are no great differences, and overall, all four batches showed excellent disintegration. The complete disintegration appears in less than one minute. At both molar ratios tested, the tablets with the physical blends showed faster disintegration than the tablets with co-crystallization compounds (36 s for F2 and 41 s for F4). Of the batches containing the co-crystallization compounds, the tablets with a 1:1 molar ratio of RIV to NIA showed faster disintegration: F1 took 40 s compared to 58 s for F3. This behavior can be explained by the higher compression forces required for F1 and F3 during the manufacturing process. In addition, preformulation studies have shown that F1 and F3 have smaller particle size, which should lead to a faster disintegration of these formulations. Nevertheless, their disintegration performance can be justified by the formation of solid bridges between particles. Solid bridges appear to have relatively strong bonds due to their structure, and tablets with these bonds may have a longer disintegration time [44].

### 3.3. In Vitro Dissolution Profiles

**Figure 7:**
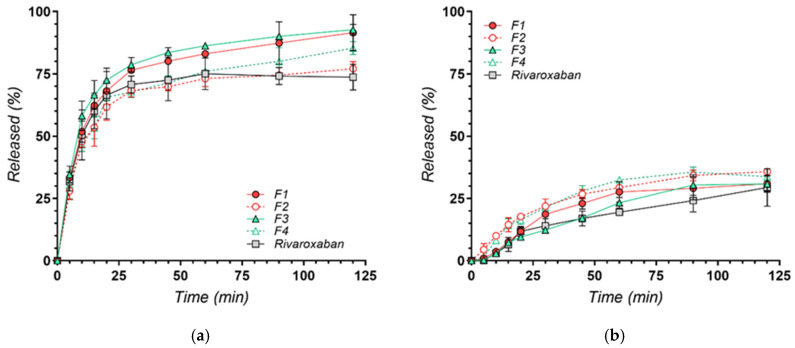
Dissolution profiles of rivaroxaban from co-crystal formulations (F1 and F3) and physical mixtures (F2 and F4) with nicotinamide, compared to pure rivaroxaban, in two different media: (**a**) pH 4.5 with 0.2% Tween 80 and (**b**) pH 6.8 phosphate buffer. Each point represents mean ± SD (n = 3).

The dissolution profiles of rivaroxaban from co-crystal formulations (F1 and F3), and their physical mixtures (F2 and F4) with nicotinamide, were comparatively evaluated against pure rivaroxaban in two standardized media commonly used for the evaluation of poorly water-soluble oral drug formulations. The pH 6.8 phosphate buffer represents a conventional simulated intestinal medium under fasted conditions, whereas the pH 4.5 acetate buffer containing surfactant corresponds to the compendial dissolution medium recommended by USP for 10 mg rivaroxaban tablets [16], providing sink conditions representative of proximal intestinal regions. The dissolution performance shows marked differences, influenced by both the formulation approach and the environmental pH conditions (Figure 7) [31].

At pH 4.5, both co-crystal formulations—F1 (1:1 molar ratio) and F3 (1:2 molar ratio)—demonstrated significantly enhanced dissolution compared to the corresponding physical mixtures (F2 and F4) and pure rivaroxaban (Figure 7a). This enhanced dissolution can be attributed to the disruption of strong intermolecular interactions within the original rivaroxaban crystal lattice by nicotinamide during co-crystallization [29]. The resulting lattice structure offers decreased lattice energy, leading to rapid initial dissolution and a supersaturated solution state. The resulting dissolution behavior aligns with the “spring and parachute” model, wherein an initial burst of dissolution (“spring”) is followed by a transient supersaturated state that can be maintained for a period (“parachute”) before recrystallization or precipitation may occur [10,45]. Additionally, the presence of Tween 80 in the slightly acidic dissolution medium improved particle wettability and dispersion, further facilitating efficient dissolution [45].

At pH 6.8, the dissolution behavior of all tested systems was markedly limited (Figure 7b). In this near-neutral medium, the overall extent of drug release remained low for all formulations, including pure rivaroxaban, and none of the tested systems exceeded approximately 30% drug release over the 120 min testing period. The limited dissolution observed at pH 6.8 is likely related to the intrinsic solubility characteristics of rivaroxaban [46,47]. As a poorly water-soluble BCS Class II drug, rivaroxaban exhibits very low equilibrium solubility in aqueous media under near-neutral conditions, reported in the literature to be approximately 5–6 µg/mL [48]. Consequently, the dissolution medium may rapidly approach saturation with respect to the drug, after which further dissolution becomes thermodynamically restricted. Under such conditions, the dissolution process becomes predominantly solubility-limited rather than diffusion-controlled, and the extent of drug release is governed primarily by the equilibrium solubility of the API.

Furthermore, the minor differences between the co-crystal formulations and the physical mixtures observed under these conditions may also reflect differences in local dissolution microenvironments or transient solid–solution equilibration processes during dissolution [49]. For co-crystals containing ionizable coformers such as nicotinamide, localized pH variations at the solid–liquid interface have been proposed in the literature as a potential factor influencing dissolution behavior [50,51].

From a biopharmaceutical perspective, this behavior is consistent with the dissolution-limited characteristics reported for rivaroxaban [52]. Considering an intrinsic aqueous solubility of approximately 5 µg/mL and a typical intestinal fluid volume of about 250 mL, the maximum amount of rivaroxaban that could theoretically dissolve under near-neutral intestinal conditions is approximately 1.25 mg. For a 10 mg dose, this corresponds to a dose number significantly greater than unity, indicating that dissolution may represent a limiting factor for drug availability in the intestinal environment [53,54].

Previous literature indicates that co-crystals incorporating nicotinamide typically exhibit higher solubility under acidic conditions due to increased protonation, which stabilizes cocrystal interactions via enhanced hydrogen bonding [7,22,50,51]. At higher pH levels, diminished protonation reduces the stability of these interactions, promoting recrystallization into less soluble forms, which explains the observed decrease in dissolution at pH 6.8 [55].

Overall, the successful preparation of rivaroxaban–nicotinamide co-crystals in the two molar ratios led to significantly improved dissolution profiles in acidic media, supporting their potential to enhance the oral bioavailability of rivaroxaban, as observed also in the literature for rivaroxaban with other coformers [10,29]. While neutral pH conditions remain challenging due to the intrinsic solubility profile of the drug, the present findings confirm that nicotinamide-based co-crystallization represents a rational and effective formulation strategy tailored to maximize rivaroxaban performance under acidic conditions relevant to the upper gastrointestinal tract. The successful development of solid forms exhibiting improved dissolution under these physiologically relevant conditions supports their further investigation as promising oral dosage forms for thromboembolic therapy.

To elucidate the potential impact of the rivaroxaban–niacinamide system on oral drug absorption, future in vivo studies and, also, long-term stability measurements will be conducted to confirm these findings.

## 4. Conclusions

This study successfully demonstrated the formation of a new rivaroxaban–niacinamide co-crystalline phase.at two distinct molar ratios (1:1 for F1 and 1:2 for F3) using the solvent evaporation method. Comprehensive physicochemical characterization through FT-IR, SEM, XRD, and TG–DTA confirmed the formation of new co-crystalline solid phases, indicating the establishment of intermolecular interactions between the drug and coformer.

The results revealed that the molar ratio has no significant influence on the structural and functional attributes of the resulting co-crystals. From a pharmacotechnical point of view, both co-crystals showed better flow and compressibility properties than the pure drug, facilitating their potential application in solid dosage formulations.

Dissolution studies revealed a marked increase in the dissolution rate of rivaroxaban from both co-crystals, compared with pure rivaroxaban. This improvement can be attributed to changes in the solid-state structure and the presence of highly soluble niacinamide, which promoted faster drug release.

In conclusion, co-crystallization with niacinamide is a promising approach for enhancing the physicochemical and biopharmaceutical properties of rivaroxaban. The findings also highlight the importance of coformer stoichiometry in tailoring co-crystal performance. These results support further development of RIV–NIC co-crystals as potential candidates for improved oral formulations of rivaroxaban.

## Figures and Tables

**Table 1:** The formulations of the tablets containing RIV-NIA co-crystallization compounds and physical mixtures.

Ingredients	Quantity (mg)/Tablet				Role in Formulation
	F1	F2	F3	F4	
RIV-NIA (1:1 co-crystallization compound)	13	-	-	-	Active ingredient
RIV-NIA (1:1 physical mixture)	-	-	13	-	Active ingredient
RIV-NIA (1:2 co-crystallization compound)	-	16	-	-	Active ingredient
RIV-NIA (1:2 physical mixture)	-	-	-	16	Active ingredient
Avicel^®^ PH 102—microcrystalline cellulose	91.5	91.5	90	90	Filler Binder
Flowlac^®^ 100—spray-dried lactose	91.5	91.5	90	90	Filler Binder
EXPLOTAB^®^—Sodium starch glycolate	2	2	2	2	Superdisintegrant
LIGAMED^®^ MF-2-V—Magnesium stearate	2	2	2	2	Glidant
TOTAL	200	200	200	200	

**Table 2:** The 2θ angles for the first five most intense peaks specific to Rivaroxaban, as calculated from COD file, fit of the observed pattern, and contribution to the fit of the co-crystal compounds.

No.	RIV-Calculated from COD # 2,242,344	RIV-Fit	RIV-Contribution for RIV-NIA 1:1	RIV-Contribution for RIV-NIA 1:2
1	22.5016	22.8568	22.7355	22.7271
2	19.928	20.2734	20.152	20.1445
3	16.5242	16.8682	16.7462	16.7374
4	19.5317	19.7252	19.7628	19.7563
5	26.6499	27.003	26.8814	26.8728

**Table 3:** The 2θ angles for the first most intense five peaks specific for niacinamide as calculated from COD file, fit of the observed pattern and contribution to the fit of the co-crystal compounds.

No.	NIA-Calculated from COD # 2,003,051	NIA-Fit	NIA-Contribution for RIV + NIA 1:1	NIA-Contribution for RIV + NIA 1:2
1	14.8289	15.0058	15.0014	14.9919
2	27.9054	27.5267	27.5186	27.5135
3	26.3875	26.062	26.0452	26.0411
4	25.868	25.5993	25.59	25.5762
5	23.875	23.5736	23.5708	23.5525

**Table 4:** Estimated crystalline phase fractions of rivaroxaban and niacinamide and crystallinity determined from Rietveld refinement of the powder XRD patterns.

	RIV-NIA (1:1 Cocrystallization Compound)		RIV-NIA (1:2 Cocrystallization Compound)	
Compound	RIV	NIA	RIV	NIA
COD #	2,242,344	2,003,051	2,242,344	2,003,051
w (%)	13.3	86.7	30	70
Crystallinity (%)	59.29		61.66	
R_exp_	8.6688		8.2865	
R_p_	9.7408		10.0004	
WR_p_	12.1403		13.3551	
χ^2^	1.9613		2.5971	

**Table 5:** Quality properties of the tablets.

Parameter	Formulation Code			
	F1	F2	F3	F4
Thickness (mm)	2.55 ± 0.06	2.52 ± 0.07	2.56 ± 0.03	2.59 ± 0.18
Diameter (mm)	10.00 ± 0.14	10.00 ± 0.21	10.00 ± 0.11	10.00 ± 0.19
Mass uniformity (mg)	200.00 ± 1.45	200.00 ± 2.32	200.00 ± 1.78	201.00 ± 2.94
Hardness (N)	78.00 ± 2.36	89.00 ± 2.88	71.00 ± 2.56	90.00 ± 2.15
Friability (%)	0.02 ± 0.01	0.05 ± 0.03	0.03 ± 0.02	0.06 ± 0.01
In vitro disintegration time (seconds)	40	36	58	41

## Data Availability

The original contributions presented in this study are included in the article. Further inquiries can be directed to the corresponding author.
